# Cost effectiveness of difelikefalin for the treatment of patients with chronic kidney disease-associated pruritus undergoing hemodialysis in Italy

**DOI:** 10.1007/s40620-024-02144-x

**Published:** 2024-11-08

**Authors:** Lucio Manenti, Andrea Marcellusi, Eugenio Di Brino, Andrea Aiello, Asia Barugolo, Patrizia Berto, Marco Soro

**Affiliations:** 1 UOC Nefrologia e Dialisi. ASL 5 Liguria, La Spezia, Italy; 2 Department of Pharmaceutical Sciences – DISFARM, University of Milan, Milan, Italy; 3 Altems Advisory, spin-off dell’Università Cattolica del Sacro Cuore, Rome, Italy; 4 PharmaLex Italy, Milan, Italy; 5 Global Value Access & Policy, CSL Vifor, Glattbrugg, Switzerland

**Keywords:** Difelikefalin, CKD-aP, Hemodialysis, Cost-effectiveness, Italy

## Abstract

**Background:**

Chronic kidney disease (CKD)-associated pruritus is a condition that strongly impacts CKD patients and is associated with increased morbidity/mortality, and worse health-related quality of life (HRQoL). Difelikefalin is currently the only drug approved in Europe specifically for treating moderate to severe CKD-associated pruritus in patients undergoing hemodialysis. The KALM-1 and KALM-2 trials showed better efficacy of difelikefalin vs placebo and best supportive care. The aim of this study was to investigate the cost-effectiveness of difelikefalin according to the Italian National Health Service (NHS) perspective.

**Methods:**

A cohort model represented by four health states (No, Mild, Moderate, and Severe pruritus) was adapted to the Italian setting. The model used data from the KALM-1 and -2 trials for efficacy, integrated with other publications for HRQoL estimations. To assess the cost of disease management, a recent Italian publication on CKD-associated pruritus was used and a price of €27 per difelikefalin vial was assumed. The base case analysis over a 15-year time horizon, and an additional 10-year scenario analysis, were established. Additionally, both deterministic univariate analysis and probabilistic multivariate sensitivity analyses were developed. Discount rates of 3% were applied. An acceptability threshold of 40,000 €/quality-adjusted life-year (QALY) was considered.

**Results:**

The results show that difelikefalin plus best supportive care is cost-effective vs best supportive care alone, with an incremental cost-effectiveness ratio, in the base case, of €35,823/QALY. Both the scenario and sensitivity analyses confirmed the strength of the results.

**Conclusions:**

Difelikefalin was found to be a cost-effective treatment for the Italian NHS. These results support its reimbursement and its inclusion in routine clinical practice.

**Graphical abstract:**

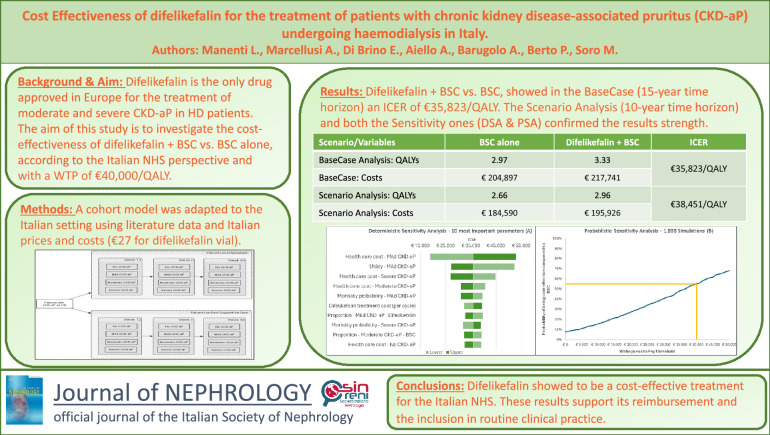

## Introduction

Chronic kidney disease (CKD)-associated pruritus is a generalized, persistent, and refractory pruritus that affects many patients with both advanced chronic kidney disease and patients on hemodialysis [1].

Data reported in the Dialysis Outcomes and Practice Patterns Study (DOPPS) showed that in Italy, about 60% of hemodialysis patients were affected by CKD-associated pruritus and 34% reported suffering from moderate to extreme itching [1].

CKD-associated pruritis can be generalized, affecting the whole body, or localized to several specific areas such as the scalp, face, upper back, arms (particularly where dialysis is performed) or groin [2].

Among hemodialysis patients, severe CKD-associated pruritus has been associated with increased risk of mortality: several studies reported a higher rate of mortality in the presence of severe or extreme pruritus, compared with patients without pruritus, with a hazard ratio of 1.24 (95% Confidence Interval: 1.08 to 1.41) [1, 3].

In addition, the most recent data show that CKD-associated pruritus, particularly in moderate and severe forms, significantly impairs patients’ HRQoL, sleep, and mood, and also increases the risk of infection-related complications and hospitalizations [4–7]. Overall, it has been observed that the physical and mental health status of patients with CKD-associated pruritus worsens as the severity of pruritus increases [4, 7, 8].

Moderate to severe CKD-associated pruritus can also have a negative economic impact both in terms of health care expenditure incurred by the National Health Service (NHS) and on the work efficiency of affected individuals, with a negative effect on society as a whole [9, 10].

Pharmacological treatments reported in the literature include the use of first- and second-generation antihistamines, corticosteroids, the κ-opioid receptor agonist nalfurafin, gabapentin, pregabalin and, sporadically, thalidomide. They are characterized by limited efficacy [11–14].

Difelikefalin is the first and only treatment indicated and approved in Europe and reimbursed in several European Union (EU) countries for the treatment of moderate to severe pruritus associated with CKD in adult patients undergoing hemodialysis. Difelikefalin, a selective κ-opioid receptor agonist, exerts its anti-inflammatory and antipruritic effects through activation of these receptors on peripheral sensory neurons and immune cells [15].

Since difelikefalin received European marketing approval (February 2022), filling the hitherto unmet need for a targeted, clinically validated treatment, updated guidelines have been published in Germany, Spain, and France recommending its use [12–14].

To date, a cost-effectiveness analysis comparing difelikefalin vs. best supportive care, from the perspective of the UK National Health Service, has been published that demonstrated cost-effectiveness of the treatment, with incremental cost-effectiveness ratios in the £20,000–£30,000 per quality adjusted life year (QALY) thresholds [16].

The Italian Medicines Agency (AIFA) assesses the place in therapy and reimbursement of drugs in Italy for the NHS. According to the Ministerial Decree dated 2 August 2019 and the AIFA Guidelines for the preparation of Dossiers for reimbursement and pricing applications, a full economic evaluation is required for new medicinal products, orphan drugs and/or new indications of already marketed active ingredients [17]. However, the details of the economic assessment remain confidential. Additionally, no published pharmacoeconomic evidence, on the cost-effectiveness of difelikefalin in the Italian setting is available. In consideration of the above, a published cost-effectiveness analysis to evaluate the value for money of difelikefalin could support the assessment and consequent decisions of both clinicians and payers.

The aim of this study was to estimate the cost-effectiveness of difelikefalin vs. best supportive care in patients with CKD-associated pruritus undergoing hemodialysis for kidney failure, from the perspective of the Italian NHS.

## Methods

A previously published cost-effectiveness model was adapted to the Italian context, in accordance with AIFA Guidelines, and validated by a panel of three independent experts, on disease/clinical features and/or pharmacoeconomic modeling [16, 17].

**Fig. 1:**
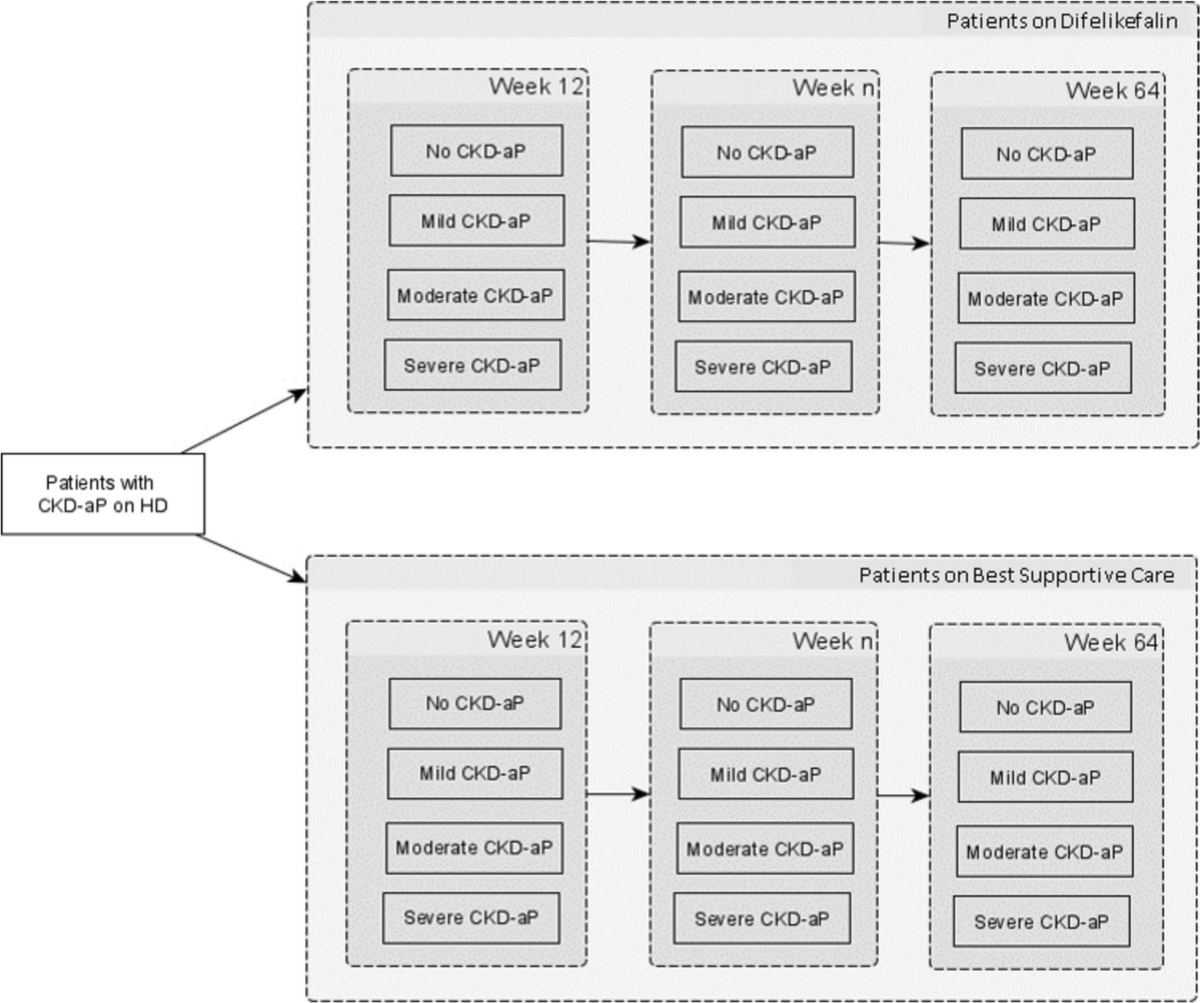
Cohort model structure. Legend: *CKD*-aP = Chronic Kidney Disease-associated Pruritus.; *HD* hemodialysis

The original model, described in detail in the specific publication and reported in Fig. 1, is a cohort model consisting of 4 health states differentiated by itch severity (No CKD-associated pruritus, mild CKD-associated pruritus, moderate CKD-associated pruritus, and severe CKD-associated pruritus) and developed to reflect the natural history of the disease [16].

In the model, the 5-D Itch Scale is used as a multidimensional tool to assess pruritus intensity and related QoL in five distinct domains (duration, degree, direction, disability, and distribution). The total score of the 5-D Itch Scale ranges from 5 to 25, with higher scores indicating worse itch intensity and worse itch-related quality of life. A reduction from baseline of ≥ 5 points in the total 5-D Itch Scale score represents a clinically significant improvement [16, 18].

This scale is composed of 5 dimensions: 1) Duration, 2) Degree, 3) Direction, 4) Disability, 5) Distribution. The aggregate score of the 5-D scale ranges from 5 to 25 points, with higher scores indicating more severe itching and a greater impact on quality of life [18].

In the first part of the model, up to 64 weeks (16 cycles: each cycle lasting 4 weeks) of clinical data from the trials were used; thereafter the model considers extrapolations over the long term, with the population varying depending on mortality associated with itch severity [1, 16, 19–23].

Data from the KALM-1 and KALM-2 studies were used to estimate efficacy for difelikefalin and the first 12 weeks of best supportive care [19, 20, 22, 23]. In both KALM studies patients who continued in the open label extension, including those who received placebo/best supportive care, switched to open-label difelikefalin after the first 12 weeks of the double-blind treatment phase [19, 20, 22, 23]. Therefore, in the post-randomization phase, data from the “SHARE-HD stepped wedge controlled randomized trial” [21] (SWCRT) were integrated for best supportive care patients to complement the lack of data from the KALM studies.

The model includes “responder” and “non-responder” categories to capture differences in treatment duration (and thus treatment costs) between these two groups. Responders are defined as subjects who achieve a pre-defined reduction in pruritus intensity, while non-responders are patients who discontinue treatment due to lack of efficacy [16].

In the analysis, patients with “severe” status after three cycles in the difelikefalin arm are assumed to discontinue difelikefalin, due to lack of response. Subjects who start with moderate severity CKD-associated pruritus and do not progress to mild or no severity model health states also discontinue difelikefalin therapy, while patients with severe pruritus at baseline who progress to a “moderate” state as a result of treatment, continue on difelikefalin therapy [16].

After the sixteenth cycle, the model predicts that patients will maintain the same health status, influenced solely by mortality data related to the severity of pruritus, as estimated from disease information in the literature (Table 1) [1].

**Table 1:** Input data analysis according to the health states

CKD-aP	No	Mild	Moderate	Severe
Mortality and Utility coefficients				
Mortality	0.0108	0.0104	0.0130	0.0144
Utility	0.62	0.58	0.47	0.37
Cost data per cycle (28 days)				
Difelikefalin	€ 324			
Best Supportive Care	€ 0			
Other Health Care Costs related to the Disease	€ 2,407	€ 2,407	€ 2,851	€ 2,851

Legend: *CKD-aP* Chronic Kidney Disease-associated Pruritus

An ordered Probit mixed-effects regression model, including age, sex, diabetes mellitus, and polynomial terms for time is used to generate observations between study sampling time points when values are needed and patient responses in the study are missing. Full model details can be found in the original publication [16].

Regarding utilities, for patients with mild to severe CKD-associated pruritus, the model was populated, in the absence of specific Italian data, with utility data from a recent French, prospective, multicenter study of 1,304 adult patients on hemodialysis, as this population was considered the best match with the Italian population [24]. Since the French study did not report a value for patients without pruritus, the value reported in the original analysis, with values rounded to the second decimal place, was considered for this health status (Table 1) [16].

Estimation of the cost of therapy (Table 1) is based on the following assumptions: a) a hypothetical placeholder price of €27 for a 50 mcg vial of difelikefalin (0.5 mcg per kg) with consumption of one vial per patient for each hemodialysis session (considering an average of 3 per week), equivalent to a cost per cycle (28 days) of €324; b) the cost of best supportive care in both model arms is assumed equal to zero.

Data from an Italian real-world administrative database analysis were used to estimate direct health care costs (drugs, hemodialysis, hospitalizations and outpatient services) as a function of disease severity. The costs for CKD patients without pruritus were used as a baseline to represent both patients without pruritus and those with mild pruritus. Conversely, the cost of treatment for CKD-associated pruritus patients was used as a proxy for both moderate itch and severe itch patients. Annual costs were divided by 13 (number of annual cycles) and rounded to the first whole number (Table 1), with a cost per cycle (28 days) of: (a) €2,407 for patients without pruritus and with mild pruritus; (b) €2,851 for patients with moderate and severe pruritus [10].

In the analysis, the cost of dialysis associated with incremental survival was deducted from the total cost, in accordance with the National Institute for Health and Care Excellence (NICE) recommendations, thus allowing correct incremental cost-effectiveness ratio estimation; not including this adjustment, the incremental survival gain provided by active treatment would have a negative effect on incremental cost-effectiveness ratios, penalizing the health technology that indeed generates additional benefit [25–27].

A 15-year time horizon is considered in the base case so that the benefits and costs of therapies under consideration could be fully captured, and an additional 10-year Scenario Analysis is also provided. These time horizons were selected according to the clinical expert’s opinion, the age of the affected patients and the life expectancy in Italy.

Two different sensitivity analyses are also provided: Univariate (DSA-Tornado), with ± 10% variance on all variables, and probabilistic sensitivity analysis with 1000 simulations [28]. Both sensitivity analyses span a time horizon of 15 years, as in the base case. An annual discount rate of 3% is applied to clinical benefits and costs in all analyses [17].

To assess the cost-effectiveness of difelikefalin vs best supportive care, incremental cost-effectiveness ratios are presented considering a willingness-to-pay threshold of €40,000/QALY. This willingness-to-pay was selected according to the threshold values identified by the Italian Health Economics Association (AIES) working group and the results of the recent publication from AIFA members on the mean incremental cost-effectiveness ratio of drugs approved by the NHS following relatively recent negotiations [29–31].

## Results

In the base case with a 15-year time horizon, the results show that upon addition of difelikefalin treatment, there is a greater quality of life weighted efficacy. In fact, for patients treated with difelikefalin/best supportive care, a total of 3.33 QALYs is estimated vs. 2.97 QALYs in the group with best supportive care alone, an increase of + 0.373 QALYs.

Analyzing therapy costs, the introduction of difelikefalin results in an increase in total costs, + €12,834 estimated as the difference between the + €21,237 total costs (€226,134 vs. €204,897, respectively, for difelikefalin/best supportive care and best supportive care alone) and a saving of €8,393 in hemodialysis costs due to incremental survival in the difelikefalin arm. This corresponds to an incremental cost-effectiveness ratio of €35,823/QALY, lower than the identified willingness-to-pay (€40,000/QALY). The 10-year scenario analysis, shows very similar values, with an incremental cost-effectiveness ratio of €38,451/QALY. It should also be noted that in both analyses, the cost of difelikefalin alone represents a small percentage (9%) of the total costs associated with the disease and its management. The results of the base case and scenario analysis are shown in Table 2.

**Table 2:** Cost-Effectiveness results: BaseCase and Scenario Analysis

	Placebo/ BSC [A]	Difelikefalin [B]	Δ [B-A]
BaseCase Analysis: Time Horizon 15 years			
Main Treatment Cost (€)	€ 0	€ 21,231	+ € 21,231
Total Health Care Costs (€)	€ 204,897	€ 204,903	+ € 6
Total Gross Cost (€)	€ 204,897	€ 226,134	+ € 21,237
Dialysis Cost due to Incremental Survival	€ 0	−€ 8393	−€ 8393
Total Net Cost (€)	€ 204,897	€ 217,741	+ € 12,844
QALYs	2.97	3.33	+ 0.36
ICER	€ 35,823/QALY		
Scenario Analysis: Time Horizon 10 years			
Main Treatment Cost (€)	€ 0	€ 18,828	+ € 18,828
Total Health Care Costs (€)	€ 184,590	€ 182,770	-€ 1,820
Total Gross Cost (€)	€ 184,590	€ 201,598	+ € 17,008
Dialysis Cost due to Incremental Survival	€ 0	-€ 5,672	-€ 5,672
Total Net Cost (€)	€ 184,590	€ 195,926	+ € 11,336
QALYs	2.66	2.96	+ 0.30
ICER	€ 38,451/QALY		

Legend:* ICER* Incremental Cost-Effectiveness Ratio*; QALY* Quality Adjusted Life Years*;* BSC Best Supportive Care

**Fig. 2:**
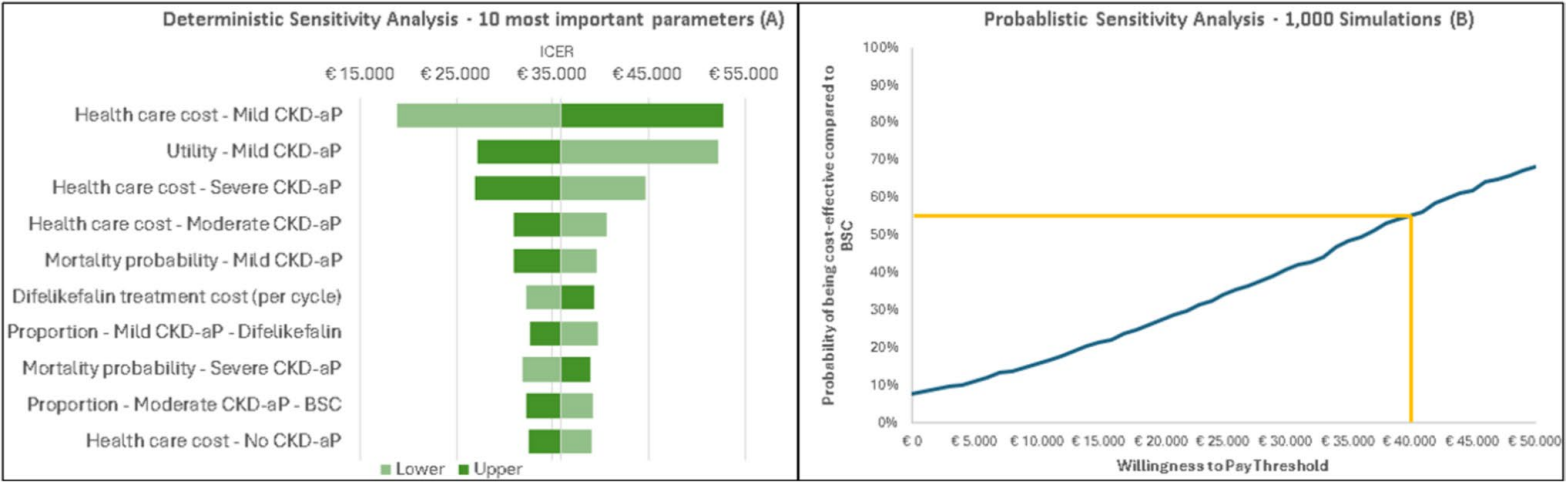
Cost-Effectiveness results: Discrete Sensitivity Analysis and Probabilistic Sensitivity Analysis. Legend*: BSC = *best supportive care; *CKD-aP* = chronic kidney disease-associated pruritus. Parameter uncertainty may be represented via deterministic sensitivity analysis (DSA) or via Probabilistic Sensitivity Analysis (PSA) [28]. In a DSA, parameter values are varied manually to test the sensitivity of the model’s results to specific parameters or sets of parameters. In a PSA, (preferably) all parameters are varied simultaneously, with multiple sets of parameter values being sampled from a priori defined probability distributions. This is repeated several times, resulting in a distribution of outputs. A key output of a PSA is the proportion of results that are considered cost-effective in relation to a given Willingness-to-Pay Threshold [28]. Willingness-to-Pay Threshold is the maximum amount a decision-maker is willing to pay for a unit of gained health outcome [31]

Deterministic sensitivity analysis shows results close to the base case incremental cost-effectiveness ratio, with greater variation associated with health care costs and utilities associated with patients with mild disease (Min: €18,808/QALY; Max: €52,832/QALY). This is probably due to the longer time and greater time differential for patients in the difelikefalin arm who remain in this health state compared with best supportive care alone (Fig. 2).

Probabilistic sensitivity analysis (out of 1,000 simulations) also confirmed the favorable cost-effectiveness profile of difelikefalin, with a 55% probability that the treatment is cost-effective, at a willingness-to-pay of €40,000/QALY (Fig. 2).

## Discussion

Difelikefalin was supported by the results of two clinical trials, KALM-1 (NCT03422653) and KALM-2 (NCT03636269), where patients were randomized to receive intravenous difelikefalin (0.5mcg/kg) or placebo 3 times a week for 12 weeks. Both arms maintained ongoing best supportive care if any. The randomization phase was then followed by a 52-week open-label extension phase.

Studies demonstrated that patients treated with difelikefalin/best supportive care achieved a statistically significant superior clinical response compared to placebo/best supportive care. This response was defined as a reduction of ≥ 3 points on the Worst Itch-Numerical Rating Scale (WI-NRS) at 24 h. Comparable safety profiles were observed between the treatment arms [19, 20, 22, 23].

Based on the KALM-1 and KALM-2 clinical trials, a cost-effectiveness analysis comparing difelikefalin vs. best supportive care, from the perspective of the UK National Health Service, has been published, showing the cost-effectiveness of the treatment, with incremental cost-effectiveness ratios in the £20,000–£30,000 per QALY thresholds [20].

The analysis presented in this manuscript is based on the model developed for the UK, whose framework has been previously validated [16].

The results of this cost-effectiveness analysis, adapted to Italy, suggest that difelikefalin may represent an advantageous therapy for the Italian NHS for the management of CKD-associated pruritus. Indeed, as in the original analysis, incremental cost-effectiveness ratios are positioned in most scenarios and simulations below a hypothetical willingness-to-pay of €40,000/QALY [16].

Noteworthy, should the costs of hemodialysis associated with incremental survival not have been deducted, the incremental cost-effectiveness ratios would have exceeded €40,000/QALY: however, as discussed elsewhere and as authoritatively established by NICE, a treatment that generates an additional benefit and whose cost is attributable not only to the cost of therapy, but also incorrectly to the cost of hemodialysis associated with increased survival, would be at a considerable disadvantage. It is also difficult to compare a treatment vs. a comparator that has zero cost, as assumed in the model for best supportive care [25–27].

Instead, the differences in QALYs (UK vs. Italy) generated by the two studies are mainly due to the different utility data used in the different settings for patients with mild to severe pruritus [24].

Further analyzing the model, and as reported in the previous publication, the analyses presented are always very conservative because patients’ outcomes in the KALM-1 and KALM-2 studies were influenced by a strong placebo effect (particularly in KALM-2), resulting in a possible underestimation of the incremental benefits associated with difelikefalin use [16]. Indeed, if KALM-1 data alone were used (where the placebo effect was less evident), lower incremental cost-effectiveness ratios would be obtained, below €30,000/QALY in the base case assumptions.

Furthermore, the cost analysis is likely to underestimate possible savings in the difelikefalin arm since the addition of the drug is assumed not to affect other expenses, such as drugs (including some currently being used off-label) whose use may be reduced in the presence of active therapy. Possible savings on other health care costs (e.g., costs associated with adverse event management) are also assumed to be zero. Treatment therefore only affects the transition from and to different health states.

The main limits of the analysis are related to: a) the uncertainty of long-term data extrapolation, which heavily relies on the 64-week trial data; b) the trial population not perfectly overlapping with the Italian population; c) the cost data extrapolated from administrative databases that may underestimate the real savings, related to the introduction of the new treatment into Italian clinical practice. Despite these critical aspects, which are quite common in many cost-effectiveness analyses, the model is based on very robust clinical input, integrating data from several clinical trials for both the difelikefalin and best supportive care arms [1, 19–23].

Finally, in their publication, the authors of the original model emphasized the importance of integrating ad hoc data, preferably representative of current clinical practice in the different countries: in this Italian adaptation, use of data from the Calabria et al. 2024 study enabled the integration of resource consumption and costs for CKD-associated pruritus management in our country, thus making the analysis particularly representative for Italy, and its results useful for public decision-makers [10].

## Conclusions

The results of our analysis suggest that treatment with difelikefalin in addition to best supportive care, if any, may improve health and HRQoL of patients on hemodialysis due to better disease management, even considering the significant placebo effect observed in the best supportive care arm. Despite the conservative assumptions used in the model and the zero-cost assumption for best supportive care, difelikefalin has been shown to be a cost-effective option from the perspective of the Italian NHS. Based on these findings, the model indicates that the use of difelikefalin should be integrated into the Italian standard clinical practice.

## Data Availability

The data underlying this article will be shared on reasonable request to the corresponding author.

## References

[1] Sukul N, Karaboyas A, Csomor PA, Schaufler T, Wen W, Menzaghi F, Rayner HC, Hasegawa T, Al Salmi I, Al-Ghamdi SMG, Guebre-Egziabher F, Ureña-Torres PA, Pisoni RL (2020) Self-reported pruritus and clinical, dialysis-related, and patient-reported outcomes in hemodialysis patients. Kidney Med 3(1):42-53.e1. 10.1016/j.xkme.2020.08.011 33604539 10.1016/j.xkme.2020.08.011 PMC7873756

[2] Mettang T, Kremer AE (2015) Uremic pruritus. Kidney Int 87(4):685–691. 10.1038/ki.2013.454 24402092 10.1038/ki.2013.454

[3] Narita I, Alchi B, Omori K, Sato F, Ajiro J, Saga D, Kondo D, Skatsume M, Maruyama S, Kazama JJ, Akazawa K, Gejyo F (2006) Etiology and prognostic significance of severe uremic pruritus in chronic hemodialysis patients. Kidney Int 69(9):1626–1632. 10.1038/sj.ki.5000251 16672924 10.1038/sj.ki.5000251

[4] Kimata N, Fuller DS, Saito A, Akizawa T, Fukuhara S, Pisoni RL, Robinson BM, Akiba T (2014) Pruritus in hemodialysis patients: results from the japanese dialysis outcomes and practice patterns study (JDOPPS). Hemodial Int 18(3):657–667. 10.1111/hdi.12158 24766224 10.1111/hdi.12158

[5] Weiss M, Mettang T, Tschulena U, Weisshaar E (2016) Health-related quality of life in haemodialysis patients suffering from chronic itch: results from GEHIS (German Epidemiology Haemodialysis Itch Study). Qual Life Res 25(12):3097–3106. 10.1007/s11136-016-1340-4 27307011 10.1007/s11136-016-1340-4

[6] Millington GWM, Collins A, Lovell CR, Leslie TA, Yong ASW, Morgan JD, Ajithkumar T, Andrews MJ, Rushbook SM, Coelho RR, Catten SJ, Lee KYC, Skellett AM, Affleck AG, Exton LS, Mohd Mustapa MF (2018) Levell NJ (2018) British Association of Dermatologists’ guidelines for the investigation and management of generalized pruritus in adults without an underlying dermatosis. Br J Dermatol 178(1):34–60. 10.1111/bjd.16117 29357600 10.1111/bjd.16117

[7] Weiner DE, Schaufler T, McCafferty K, Kalantar-Zadeh K, Germain M, Ruessmann D, Morin I, Menzaghi F, Wen W, Ständer S (2023) Difelikefalin improves itch-related sleep disruption in patients undergoing haemodialysis. Nephrol Dial Transplant. 10.1093/ndt/gfad245 37968132 10.1093/ndt/gfad245 PMC11210984

[8] van der Willik EM, Lengton R, Hemmelder MH, Hoogeveen EK, Bart HAJ, van Ittersum FJ, Ten Dam MAGJ, Bos WJW, Dekker FW, Meuleman Y (2022) Itching in dialysis patients: impact on health-related quality of life and interactions with sleep problems and psychological symptoms-results from the RENINE/PROMs registry. Nephrol Dial Transplant 37(9):1731–1741. 10.1093/ndt/gfac022 35098998 10.1093/ndt/gfac022 PMC9395377

[9] Rayner HC, Larkina M, Wang M, Graham-Brown M, van der Veer SN, Ecder T, Hasegawa T, Kleophas W, Bieber BA, Tentori F, Robinson BM, Pisoni RL (2017) International comparisons of prevalence, awareness, and treatment of pruritus in people on hemodialysis. Clin J Am Soc Nephrol 12(12):2000–2007. 10.2215/CJN.03280317 28923831 10.2215/CJN.03280317 PMC5718267

[10] Calabria S, Manenti L, Ronconi G, Piccinni C, Dondi L, Dondi L, Pedrini A, Esposito I, Addesi A, Aucella F, Martini N (2024) Italian healthcare resource consumption for patients on hemodialysis treated for chronic kidney disease-associated pruritus (CKD-aP). Glob Reg Health Technol Assess 11:22–30. 10.33393/grhta.2024.2696 38234332 10.33393/grhta.2024.2696 PMC10792387

[11] Weisshaar E, Szepietowski JC, Dalgard FJ, Garcovich S, Gieler U, Giménez-Arnau AM, Lambert J, Leslie T, Mettang T, Misery L, Şavk E, Streit M, Tschachler E, Wallengren J, Ständer S (2019) European S2k Guideline on Chronic Pruritus. Acta Derm Venereol 99(5):469–506. 10.2340/00015555-3164 30931482 10.2340/00015555-3164

[12] Ständer S, Zeidler C, Augustin M, Darsow U, Kremer AE, Legat FJ, Koschmieder S, Kupfer J, Mettang T, Metz M, Nast A, Raap U, Schneider G, Ständer H, Streit M, Schut C, Weisshaar E (2022) S2k Leitlinie: Diagnostik und Therapie des chronischen Pruritus. J Dtsch Dermatol Ges 20(10):1386–1402. 10.1111/ddg.14830_g 36252075 10.1111/ddg.14830_g

[13] Buades JM, Figueras-Nart I, Goicoechea M, Sánchez Villanueva RJ, Serra-Baldrich E, en representación del Grupo Español de Prurito Asociado a ERC (2023) Documento de información y consenso para el manejo diagnóstico y terapéutico del prurito asociado a la enfermedad renal crónica en pacientes en hemodiálisis en España. Nefrologia. 10.1016/j.nefro.2023.04.00638182446

[14] Lanot A, Misery L, Rostoker G, Testa A, Chauveau P, Touzot M, Florens N, Bataille P (2024) Diagnostic et prise en charge du prurit associé à la maladie rénale chronique chez les patients hémodialysés [Diagnosis and management of pruritus associated with chronic kidney disease in hemodialyzed patients]. Nephrol Ther 20(1):50–60. 10.1684/ndt.2024.60 38294264 10.1684/ndt.2024.60

[15] European Medicine Agency. Kapruvia (difelikefalin). (2022). Kapruvia | European Medicines Agency (europa.eu) Accessed 6 Apr 2024.

[16] Thokala P, Hnynn Si PE, Hernandez Alava M, Sasso A, Schaufler T, Soro M, Fotheringham J (2023) Cost effectiveness of difelikefalin compared to standard care for treating chronic kidney disease associated pruritus (CKD-aP) in people with kidney failure receiving haemodialysis. Pharmacoeconomics 41(4):457–466. 10.1007/s40273-022-01237-4 36735201 10.1007/s40273-022-01237-4 PMC10020261

[17] Agenzia Italiana del Farmaco (2020) Linee guida per la compilazione del dossier a supporto della domanda di rimborsabilità e prezzo i un medicinale. https://www.aifa.gov.it/documents/20142/1283800/Linee_guida_dossier_domanda_rimborsabilita.pdf Accessed 12 Apr 2024.

[18] Elman S, Hynan LS, Gabriel V, Mayo MJ (2010) The 5-D itch scale: a new measure of pruritus. Br J Dermatol 162(3):587–593. 10.1111/j.1365-2133.2009.09586.x 19995367 10.1111/j.1365-2133.2009.09586.x PMC2875190

[19] Wooldridge TD, McCafferty K, Schoemig M, Csiky B, Zwiech R, Wen W, Munera C, Menzaghi F (2020) Efficacy and safety of difelikefalin for moderate-to-severe CKD–associated pruritus: a global phase 3 study in hemodialysis patients (KALM-2). American Society of Nephrology Kidney Week Virtual conference https://www.caratherapeutics.com/wp-content/uploads/2022/05/Woolridge-T-et-al.-Presented-at-the-American-Society-of-Nephrology-Kidney-Week-2020-1.pdf Accessed 7 Apr 2024.

[20] Fishbane S, Jamal A, Munera C, Wen W, Menzaghi F, KALM-1 Trial Investigators (2020) A phase 3 trial of difelikefalin in hemodialysis patients with pruritus. N Engl J Med 382(3):222–232. 10.1056/NEJMoa1912770 31702883 10.1056/NEJMoa1912770

[21] Fotheringham J, Barnes T, Dunn L, Lee S, Ariss S, Young T, Walters SJ, Laboi P, Henwood A, Gair R, Wilkie M (2020) A breakthrough series collaborative to increase patient participation with hemodialysis tasks: a stepped wedge cluster randomised controlled trial. PLoS ONE 16(7):e0253966. 10.1371/journal.pone.0253966 10.1371/journal.pone.0253966 PMC8291659 34283851

[22] Topf J, Wooldridge T, McCafferty K, Schömig M, Csiky B, Zwiech R, Wen W, Bhaduri S, Munera C, Lin R, Jebara A, Cirulli J, Menzaghi F (2022) Efficacy of difelikefalin for the treatment of moderate to severe pruritus in hemodialysis patients: pooled analysis of KALM-1 and KALM-2 phase 3 studies. Kidney Med 4(8):100512. 10.1016/j.xkme.2022.100512 36016762 10.1016/j.xkme.2022.100512 PMC9396406

[23] Fishbane S, Wen W, Munera C, Lin R, Bagal S, McCafferty K, Menzaghi F, Goncalves J (2022) Safety and tolerability of difelikefalin for the treatment of moderate to severe pruritus in hemodialysis patients: pooled analysis from the phase 3 clinical trial program. Kidney Med 4(8):100513. 10.1016/j.xkme.2022.100513 36039153 10.1016/j.xkme.2022.100513 PMC9418597

[24] Lanot A, Bataille S, Rostoker G, Bataille P, Chauveau P, Touzot M, Misery L (2023) Moderate-to-severe pruritus in untreated or non-responsive hemodialysis patients: results of the French prospective multicenter observational study Pruripreva. Clin Kidney J 16(7):1102–1112. 10.1093/ckj/sfad032 37398693 10.1093/ckj/sfad032 PMC10310516

[25] Davis S, Assessing Technologies That Are Not Cost-Effective at a Zero Price (2014) National Institute for Health and Care Excellence (NICE). London 27466661

[26] Davis S, Akehurst R (2016) How Do We Evaluate Technologies That Are Not Cost Effective at Zero Price? Value & Outcomes Spotlight. vos-cost-effective.pdf (ispor.org) Accessed 14 Apr 2024

[27] National Institute for Health and Care Excellence (2018) Guideline NG107. Renal replacement therapy and conservative management. https://www.nice.org.uk/guidance/ng107/evidence/b-modalities-of-rrt-pdf-6542344047?mc_phishing_protection_id=28632-cg102hdse2iscje1fl1g Accessed 15 Apr 2024. 31194310

[28] Briggs AH et al (2012) Model parameter estimation and uncertainty: a report of the ISPOR-SMDM modeling good research practices task force-6. Value in Health 15(6):835–842 22999133 10.1016/j.jval.2012.04.014

[29] Fattore G, (2009) Proposta di linee guida per la valutazione economica degli interventi sanitari in Italia. Pharmacoeconomics-Ital-Res-Articles 11: 83–93. 10.1007/BF03320660.

[30] Russo P, Zanuzzi M, Carletto A, Sammarco A, Romano F, Manca A (2023) Role of economic evaluations on pricing of medicines reimbursed by the italian national health service. Pharmacoeconomics 41(1):107–117. 10.1007/s40273-022-01215-w 36434415 10.1007/s40273-022-01215-w PMC9813158

[31] Willingness-to-Pay [online]. (2016). York; York Health Economics Consortium; 2016. https://yhec.co.uk/glossary/willingness-to-pay/. Accessed 19 Sept 2024

